# *Daphnia* diversity on the Tibetan Plateau measured by DNA taxonomy

**DOI:** 10.1002/ece3.4071

**Published:** 2018-04-24

**Authors:** Lei Xu, Qiuqi Lin, Shaolin Xu, Yangliang Gu, Juzhi Hou, Yongqin Liu, Henri J. Dumont, Bo‐Ping Han

**Affiliations:** ^1^ South China Sea Fisheries Research Institute Chinese Academy of Fishery Sciences Guangzhou China; ^2^ Institute of Hydrobiology Jinan University Guangzhou China; ^3^ Institute of Tibetan Plateau Research Chinese Academy of Sciences Beijing China; ^4^ Guangdong Provincial Key Laboratory of Fishery Ecology and Environment Guangzhou China; ^5^ Key Laboratory of South China Sea Fishery Resources Development and Utilization Ministry of Agriculture Guangzhou China

**Keywords:** COI, *Daphnia*, DNA taxonomy, Tibetan Plateau

## Abstract

*Daphnia* on the Tibetan Plateau has been little studied, and information on species diversity and biogeography is lacking. Here, we conducted a 4‐year survey using the barcoding fragment of the mitochondrial COI gene to determine the distribution and diversity of *Daphnia* species found across the Plateau. Our results show that species richness is higher than previously thought, with total described and provisional species number doubling from 5 to 10. Six of the taxonomic units recovered by DNA taxonomy agreed well with morphology, but DNA barcoding distinguished three clades each for the *D. longispina* (*D. galeata*,* D. dentifera,* and *D. longispina*) and *D. pulex* (*D. pulex*,* D*. cf. *tenebrosa,* and *D. pulicaria*) complexes. The sequence divergence between congeneric species varied within a large range, from 9.25% to 30.71%. The endemic *D. tibetana* was the most common and widespread species, occurring in 12 hyposaline to mesosaline lakes. The lineage of *D. longispina* is the first confirmed occurrence in west Tibet.

## 1 INTRODUCTION

In the past decade, DNA sequencing has generated abundant molecular information, standard dataset platforms, and universal technical rules for modern taxonomic and biogeographical research (Ratnasingham & Hebert, 2007). DNA barcoding uses a short DNA sequence in an organism's DNA to compare against that of another organism to determine the degree of relatedness between two closely related organisms. The barcoding fragment of the mitochondrial gene cytochrome *c* oxidase subunit I (COI) is a popular marker used to identify and differentiate closely related species that are very similar in morphology. It has assisted in species‐level identity in many animal groups such as birds (Hebert, Stoeckle, Zemlak, & Francis, 2004), fishes (Ward, Zemlak, Innes, Last, & Hebert, 2005), spiders (Barrett & Hebert, 2005), butterflies (Hebert, Cywinska, Ball, & deWaard, 2003; Janzen et al., 2005), ants (Smith, Fisher, & Hebert, 2005), and crustaceans (Costa et al., 2007; Elías‐Gutiérrez, Jerónimo, Ivanova, Valdez‐Moreno, & Hebert, 2008), including marine decapods and euphausiids (Bucklin et al., 2007; Costa et al., 2007).

Cladocera is a monophyletic, primarily freshwater crustacean order, one of the three main components of the microcrustacean zooplankton (Dumont & Negrea, 2002). The genus *Daphnia* (Anomopoda: Daphniidae) has been studied in much detail (Lampert, 2011), and the full genome of two species (*D. magna* and *D. pulex*) has been sequenced (Colbourne, Singan, & Gilbert, 2005; Colbourne et al., 2011). *Daphnia* is most diverse and abundant in the temperate regions, but is present in all climate zones on all continents, and is often the dominant group in freshwater zooplankton (Benzie, 2005). Despite this, the taxonomy of the group remains uncertain because, inter alia, of a highly variable morphology that can be strongly modified by environmental conditions. Recently, molecular data have confirmed that some *Daphnia* cannot be identified to species by morphological means and its specific diversity remains underestimated. Analysis of sequences of mitochondrial COI and 12S rDNA genes by Hebert, Witt, and Adamowicz (2003b) revealed five phylogroups with more than 3% divergence in *D. ambigua*. Penton, Hebert, and Crease (2004) discriminated two cryptic species within the *D. obtusa* complex in North America. De Gelas and De Meester (2005) reported that populations of *D. magna* showed little COI divergence within Europe, but deep divergence was recovered in North American populations. So far in China, no barcode studies assessed *Daphnia* species diversity.

The Tibetan Plateau is widely considered as a large natural experimental area for speciation and evolution. It is the world's highest and largest plateau and is surrounded by mountain ranges that source several of the longest rivers in Asia. The Plateau supports a variety of ecosystems that harbor an exceptionally diverse flora with about 4,385 species in 1,174 genera in 189 families (Wu, 1980). More than 25% of the total species identified are endemic (Wu, 1987). This reflects the age of the plateau, the central part of which started rising some 40 million years ago. Previous fragmentary taxonomic studies of Cladocera on the Tibetan Plateau including the genus *Daphnia* were based solely on morphology (Chiang, 1963; Chiang & Du, 1979; Shen & Sung, 1964). These were updated after several scientific expeditions to the area during the 1970s (Chiang & Chen, 1974; Chiang, Shen, & Gong, 1983). More recently, Möst et al. (2013) and Ma et al. (2015) used DNA sequences to study species diversity in the region. Their studies focused on the *D. longispina* complex that is often the dominant *Daphnia* taxa in freshwater lakes and ponds found in the northern temperate region. However, Möst et al.'s sampling sites covered only two alpine lakes in the Pamir and Himalaya mountains, and Ma et al.'s study were confined to just five Tibetan lakes. A more comprehensive coverage is required in this ecologically important region of the world.

In this study, we employed DNA barcoding and DNA taxonomy through analysis of the mitochondrial marker COI to determine species diversity of the *Daphnia* genus in lakes and ponds on the Tibetan Plateau. We also estimated the number of endemic species in the region and generated a phylogenetic tree based on our mtCOI data and those from GenBank. Our study will greatly improve our understanding of distribution and species diversity in Cladocera and may have important implications for the conservation of the Tibetan Plateau freshwater fauna.

## 2 MATERIALS AND METHODS

### 2.1 Sample collection

**Figure 1:**
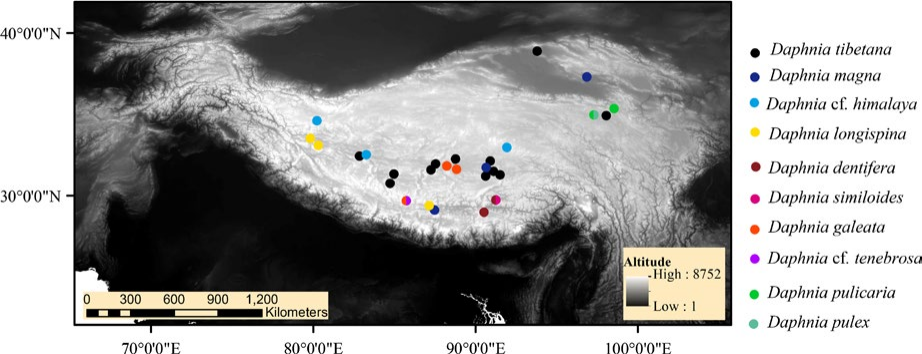
Geographic location of sample collection sites. Color dots: lakes or ponds inhabited by *Daphnia* species. The background map was generated using SRTM 90 m elevation data

Sample collection covered a large geographical range (>2,200,000 km^2^) in different habitats that ranged from 2,700 m to about 5,000 m a.s.l. Zooplankton samples were collected between 2012 and 2015 from 26 permanent lakes and from several riparian temporary ponds (Table 1 and Figure 1). Samples were obtained by vertical hauls with a plankton net that has a mesh size of 100 μm. The collected samples were fixed in 70% ethanol. Specimens were examined under a dissecting microscope in the laboratory. Sorted individuals were transferred to a fresh tube and preserved in 95% ethanol at 4°C for genetic analysis. We followed Benzie (2005) for species identification and nomenclature of Daphniidae.

**Table 1:** Geographic and environmental data for lakes and ponds where *Daphnia* populations were sampled

Lake and pond	Latitude (N)	Longitude (E)	Altitude (m)	Depth (m)	pH	Area (km^2^)	Salinity (g/L)	Predators	Nm^a^	Morphological type
Biezuoze Co	32.429	82.933	4,407	2	8.97	33	27.5	Absence	3	*D. tibetana*
Dawa Co	31.233	84.967	4,628	2	9.30	114	19.2	Absence	3	*D. tibetana*
Dajiamang Co	29.650	85.733	5,069	7	8.69	9	0.1	Presence	6	*D. galeata*;* D. *cf. *tenebrosa*
Dong Co	32.183	88.733	4,398	2	8.81	88	46.2	Absence	3	*D. tibetana*
Dagze Co	31.883	87.533	4,470	34	9.96	245	17.0	Absence	3	*D. tibetana*
Gemang Co	31.583	87.283	4,610	48	9.72	61	6.5	Presence	3	*D. tibetana*
Jiang Co	31.533	90.816	4,603	20	9.29	41	14.1	Absence	3	*D. tibetana*
Nairiping Co	31.300	91.467	4,529	7	9.98	90	8.0	Absence	3	*D. tibetana*
Peng Co	31.533	90.967	4,534	6	9.91	175	8.5	Absence	3	*D. tibetana*
Sugan Lake	38.867	93.850	2,796	4	8.90	120	20.0	Absence	2	*D. tibetana*
Youbu Co	30.783	84.800	4,645	34	9.62	64	16.0	Absence	3	*D. tibetana*
Zigetang Co	32.067	90.867	4,573	15	10.0	191	13.5	Absence	3	*D. tibetana*
Pond near Lhasa river	29.684	91.317	3,740	0.3	NA	0.01	0.8	Presence	9	*D. dentifera*;* D. similoides*
Angrenjin Co	29.206	87.390	4,304	15	9.66	24	5.3	Presence	3	*D. magna*
Bangong Co	33.500	79.841	4,241	24	8.74	604	0.5	Presence	3	*D. longispina*
Chen Co	28.967	90.533	4,436	26	8.62	40	0.8	Presence	1	*D. dentifera*
Darebu Co	32.467	83.217	4,438	3	9.40	21	1.4	Presence	3	*D*. cf. *himalaya*
Daru Co	31.667	90.750	4,688	9	9.23	70	5.1	Presence	3	*D. magna*
Dongjicuona Lake	35.283	98.567	4,086	93	8.76	232	0.4	Presence	3	*D. pulicaria*
Keluke lake	37.286	96.897	2,817	13	8.50	57	0.7	Presence	3	*D. magna*
Lang Co	29.305	87.200	4,296	26	9.44	12	1.6	Presence	3	*D. longispina*
Qiagui Co	31.817	88.300	4,558	50	8.71	88	0.2	Presence	3	*D. galeata*
Songmuxi Co	34.600	80.250	5,057	8	8.50	27	0.3	Presence	3	*D*. cf*. himalaya*
Pond near Tanggula Pass	32.904	91.951	5,142	0.3	8.54	0.01	1.9	Presence	6	*D*. cf*. himalaya*
Zhuoyang Co	34.850	98.131	4,271	7	9.23	6	7.0	Absence	3	*D. tibetana*
Zhaling Lake	34.931	97.311	4,285	13	7.70	526	0.6	Presence	5	*D. pulicaria*;* D. pulex*
Co Ngoin Lake	31.600	88.776	4,568	27	8.79	253	0.2	Presence	3	*D. galeata*
Zongxiong Co	33.100	80.283	4,351	1	8.69	9	0.2	Presence	3	*D. longispina*

a Nm = sample size for mitochondrial analysis.

### 2.2 DNA extraction

Total genomic DNA was extracted using a genomic DNA isolation kit (Wizard^®^ Genomic DNA, Purification Kit type A1225; Promega, USA). We modified the standard protocol as follows: for DNA extraction, specimens were picked out from 95% ethanol, rinsed with double‐distilled water, transferred individually to a reaction tube and stored on ice. Next, we added 200 μl warm Cell Lysis solution and 3 μl Proteinase K (20 mg/ml). We vortexed and subsequently incubated the mixture for 2 hr at 65°C and then for 2–3 days at 55°C with daily addition of 2 μl of fresh Proteinase K. Next, we added 100 μl of Precipitation Solution, vortexed vigorously at middle speed for 20 s and put on ice for 2 min, then centrifuged at RCF 15,321 *g* for 10 min at room temperature. We carefully removed the supernatant and transferred it to a clean 500 μl microcentrifuge tube containing 200 μl of isopropanol at room temperature. We centrifuged at RCF 15,321 *g* for 1 min at room temperature and carefully decanted the supernatant. Finally, the pellet was washed with 70% ethanol, dissolved in 40 μl DNA hydration solution and stored at −20°C.

### 2.3 Amplification and sequencing of the mitochondrial gene

The barcoding fragment of the mitochondrial cytochrome oxidase I (COI) gene was amplified from total genomic DNA using polymerase chain reaction (PCR) (Hajibabaei et al., 2005). Primers used for PCR were CO1490F and CO2198R (Folmer, Black, Hoeh, Lutz, & Vrijenhoek, 1994). Each 50 μl PCR reaction consisted of 31.25 μl dd H_2_O, 5 μl PCR buffer, 5 μl Coralload concentrate, 4 μl of 25 μmol/L MgCl_2_, 1 μl of 10 μmol/L dNTPs, 0.5 μl of 25 μmol/L solution of each primer, 2.5 μl DNA template, and 0.25 μl TopTaq DNA polymerase (QIAGEN, Germany). The PCR conditions for amplification were as follows: 40 cycles set at 30 s at 96°C (denaturation), 30 s at 51°C (annealing), and 60 s at 72°C (extension), followed by 7 min at 72°C (final‐extension) on a 2720 Thermal Cycler (Applied Biosystems, USA). We also used a set of primers specific for zooplankton (Prosser, Martínez‐Arce, & Elías‐Gutiérrez, 2013) for samples that responded unsuccessfully with Folmer primers. The PCR products were sequenced on an ABI 3130XL automatic sequencer. Whenever possible, we sequenced at least three individuals of each species from each population.

### 2.4 DNA taxonomy

The authenticity of all mitochondrial COI sequences was verified by a BLAST search in GenBank. The sequences were assembled and edited in BioEdit (Hall, 1999) and aligned using the CLUSTALW multiple algorithm. The first 20 and last 10 bp were not included because they were missing in some sequences. We added the COI sequences available in public databases to our analysis to ensure that our nomenclature is reliable for each *Daphnia* species (see Table S1). We used two different approaches to identify taxonomic units from DNA taxonomy, namely the Automatic Barcode Gap Discovery (ABGD) (Puillandre, Lambert, Brouillet, & Achaz, 2012) and Generalized Mixed Yule Coalescent (GMYC) model (Fujisawa & Barraclough, 2013; Pons et al., 2006) to infer putative species boundaries on COI dataset. The ABGD approach tests for a gap in the distribution of the pairwise genetic distances and then identifies groups of individuals united by genetic distances that are shorter than the gap. The method was performed on the COI alignment through an online tool (http://wwwabi.snv.jussieu.fr/public/abgd/abgdweb.html) with default settings with P (prior limit to intraspecific diversity) ranged between 0.001 and 0.1 and X (gap widths) = 1 using the available models JC86 (Jukes‐Cantor) and K80 (Kimura). The GMYC uses a maximum likelihood approach to optimize the shift in the branching patterns of the gene tree from interspecific branches (Yule model) to intraspecific branches (neutral coalescent), thereby identifies clusters of sequences corresponding to independently evolving entities. The ultrametric tree with terminals representing haplotypes, which are needed for the GMYC method, was reconstructed using BEAST1.8.0 (Drummond, Suchard, Xie, & Rambaut, 2012). Parameters for BEAST were set in BEAUti 1.8.0 assuming coalescent model with constant population size, uncorrelated relaxed clock model, general time reversible (GTR) substitution model, and gamma shape site model with a chain length of 100,000,000 iterations for Markov chain Monte Carlo (MCMC). The GMYC model was performed with the R package *splits* version 1.0‐19 (Ezard, Fujisawa, & Barraclough, 2009).

### 2.5 Genetic divergence and phylogenetic analysis

Distances between COI sequences were calculated using the Kimura two‐parameter (K2P) substitution model in MEGA, version 6 (Kumar, Nei, Dudley, & Tamura, 2008). We used uniform rates, and standard error estimates were obtained by a neighbor‐joining (NJ) bootstrap procedure with 10,000 replicates. Before phylogenetic analysis, we used MrModeltest v.2.3 (Nylander, 2004) to select the best‐fitting models of nucleotide substitution under the Akaike information criterion (AIC). Analyses were performed using Bayesian inference and maximum likelihood. Bayesian analysis was performed using MrBayes v.3.1.2 (Huelsenbeck & Ronquist, 2001; Huelsenbeck, Ronquist, Nielsen, & Bollback, 2001). The MCMC analysis was run in four parallel chains for 2,000,000 generations, sampling every 1,000 generations. For maximum likelihood analysis, we used PhyML 3.0 (Guindon et al., 2010), assuming a GTR model (Lanave, Preparata, Saccone, & Serio, 1984) with four gamma‐distributed rate categories, as suggested by ModelGenerator 0.851 (Keane, Creevey, Pentony, Naughton, & Mclnerney, 2006). We used NNI moves for tree topology searching and fast likelihood‐based parameter aLRT SH‐like for branch support. Majority rule consensus trees were reconstructed after discarding the burn‐in of 500 and displayed with treeview v.1.6.6.

## 3 RESULTS

### 3.1 Morphological survey and DNA taxonomy

**Figure 2:**
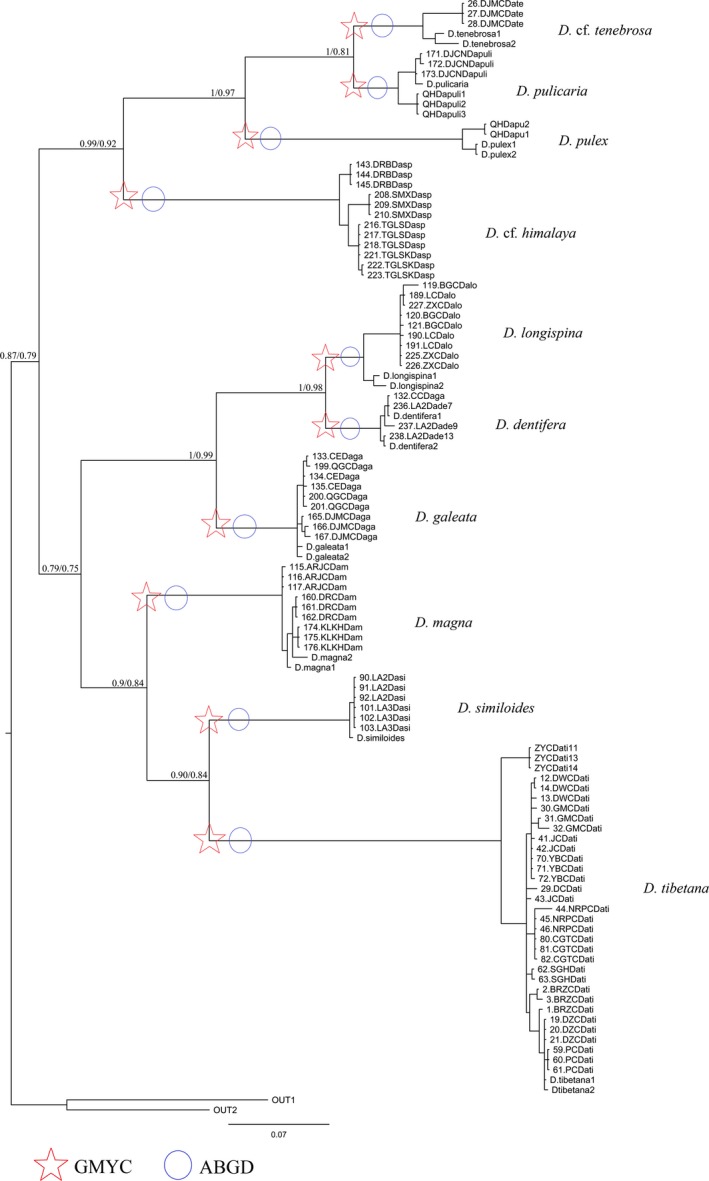
COI phylogenetic tree for *Daphnia* in Tibetan Plateau obtained from MrBayes, with the scale bars proportional to substitution rates; support values are Bayesian Posterior Probabilities support/Maximum Likelihood; ML supports are for the clades present also in the ML trees; support values below 0.7 and for short branches are not shown. The results of ABGD are shown as blue open circles and those of GMYC as red open stars on the branches

Our survey identified six morpho‐species or complexes: *D. tibetana*,* D. similoides*,* D. magna*,* D. longispina* complex, *D*. cf. *himalaya,* and *D. pulex* complex. The most common morpho‐species was *D. tibetana*, found in 12 water bodies, followed by *D. longispina* complex (found in eight water bodies); *D. magna*, and *D. pulex* complex *(*found in three water bodies). *Daphnia* cf. *himalaya* was also found in three water bodies: two permanent and one temporary. The rarest species were *D. similoides* and *D. pulex*, found only in one population each (Table 1). Sequences of COI were obtained from 93 animals (GenBank accession numbers MG544001–MG544093). Comparing each of our COI sequences with sequences in GenBank, we identified all animals as *Daphnia* (sequence divergence < 5%). The ABGD model detected a barcode gap in the alignment and suggested that the 93 individuals included 10 taxonomic units (named S1–S10). The GMYC model also supported the scenario that all analysed individuals represented 10 taxonomic units (confidence interval: 8–11). The likelihood of the null model of only one species (likelihood = 721.29) was significantly worse (likelihood ratio test = 12.73, *P* = 0.001) than that with more than one species (likelihood = 727.66). Six of the 10 taxonomic units matched morphology well. However, the *D. longispina* complex was split up into three clades—*D. galeata*,* D. dentifera* and *D. longispina*—and the *D. pulex* complex also split up into three clades—*D. pulex*,* D*. cf. *tenebrosa,* and *D. pulicaria* (Figure 2).

### 3.2 Patterns of genetic divergence and phylogenetic analysis

The total length of the sequenced segment after alignment was 677 bp. The average base composition was A = 21.70%, C = 20.41%, G = 22.36%, T = 35.53%, and transition/transversion (ti/tv) ratio = 1.751. The uncorrected K2P pairwise distances among species in this study varied between 9.25% and 30.71% and the average pairwise distance was 25.23%. The highest distance was between *D*. cf. *tenebrosa* and *D. magna*, a value which is slightly higher than the maximum congeneric distance of 30.65% recorded earlier in *Daphnia* by Costa et al. (2007). Two species of the *D. longispina* complex, viz. *D. longispina,* and *D. dentifera*, recorded the lowest distance. The uncorrected K2P pairwise distances within species varied between 0% and 1.72%. High pairwise distances within species, found in *D. tibetana* and *D. pulicaria*, reached 1.60% and 1.72%, respectively (Table 2).

**Table 2:** Genetic diversity, assessed by Kimura two‐parameter distance (median, in %) within/between the ten taxonomic units of *Daphnia* with uniform rates; standard error estimates obtained by neighbor‐joining bootstrap procedure with 10,000 replicates

Species^a^	*D. tibetana*	*D*. cf*. tenebrosa*	*D. similoides*	*D. magna*	*D. longispina*	*D. dentifera*	*D. galeata*	*D*. cf*. himalaya*	*D. pulicaria*	*D. pulex*
*D. tibetana* (12/35)	1.60 ± 0.25									
*D*. cf. *tenebrosa* (1/3)	28.77 ± 2.44	0.00								
*D. similoides* (1/3)	23.68 ± 2.15	27.56 ± 2.39	0.00							
*D. magna* (3/9)	24.64 ± 2.19	30.71 ± 2.61	17.02 ± 1.67	0.56 ± 0.21						
*D. longispina* (3/9)	29.22 ± 2.48	28.06 ± 2.43	25.63 ± 2.24	25.93 ± 2.24	0.38 ± 0.12					
*D. dentifera* (2/7)	29.20 ± 2.51	27.97 ± 2.52	26.05 ± 2.33	26.81 ± 2.46	9.25 ± 1.13	0.55 ± 0.21				
*D. galeata* (3/9)	28.75 ± 2.47	27.72 ± 2.36	25.56 ± 2.25	21.50 ± 1.91	17.24 ± 1.67	16.50 ± 1.78	0.68 ± 0.21			
*D*. cf. *himalaya* (3/12)	26.71 ± 2.36	26.64 ± 2.34	22.95 ± 2.13	26.60 ± 2.15	27.40 ± 2.27	27.19 ± 2.39	25.18 ± 2.16	1.46 ± 0.29		
*D. pulicaria* (2/6)	27.72 ± 2.38	11.39 ± 1.35	24.08 ± 2.15	28.14 ± 2.34	25.31 ± 2.21	25.14 ± 2.24	24.90 ± 2.12	24.81 ± 2.11	1.72 ± 0.41	
*D. pulex* (1/2)	29.83 ± 2.48	24.37 ± 2.19	24.99 ± 2.20	28.04 ± 2.34	27.99 ± 2.42	27.54 ± 2.44	26.73 ± 2.41	30.47 ± 2.56	23.55 ± 2.15	0.00

a Number of lakes and individuals per taxonomic unit were showed in the head column (lakes/individuals).

The best‐fitting model selected by MrModeltest 2.3 was GTR+I+G with a relative AIC weight of 0.982 and gamma distribution shape parameter 1.556. Two species of *Simocephalus* (KF484574 and KF960069) were used as outgroups to root the phylogenetic trees. Phylogenetic calculations (Bayesian inference and ML) resulted in trees of similar topology (Figure 2). COI phylogenetic tree revealed six well‐supported main clades: *D. tibetana*,* D. similoides*,* D. magna*,* D. longispina* complex, *D*. cf. *himalaya,* and *D. pulex* complex. Clade *D. longispina* complex contains three distinct genetic clusters: *D. longispina*,* D. galeata,* and *D. dentifera*. The *D. pulex* complex also contains three well‐supported sublineages: *D. pulex*,* D*. cf. *tenebrosa*, and *D. pulicaria*.

## 4 DISCUSSION

### 4.1 Species diversity and genetic divergence

Cladocera have been traditionally regarded as cosmopolitan, but there is mounting evidence for the existence of numerous sibling and cryptic species. In the past 10 years, DNA barcoding has accumulated much molecular information in support of this idea. Among examples on cladocerans, Elías‐Gutiérrez et al. (2008) applied COI barcoding to show that in Mexico and Guatemala, five species can be distinguished in the *Diapahanosoma birgei* group, while two or three taxa each were identified for *Ceriodaphnia* cf. *rigaudi*, and *Moina* cf. *micrura*. Xu et al. (2011) reconstructed the phylogeographic history of the Holarctic carnivorous cladoceran *Leptodora* and uncovered at least three species in this previously monotypic genus. In Australia, Sharma and Kotov (2013) identified three sibling species in the *Ceriodaphnia cornuta* complex. Other studies have revealed deep genetic divergences among allopatric populations of single species (De Gelas & De Meester, 2005; Thielsch, Brede, Petrusek, de Meester, & Schwenk, 2009). Recently, DNA barcoding was used to document cryptic speciation and species diversity in the sub‐Arctic region of Canada (Jeffery, Elías‐Gutiérrez, & Adamowicz, 2011), Mexico (Elías‐Gutiérrez & Valdez‐Moreno, 2008; Quiroz‐Vázquez & Elías‐Gutiérrez, 2009), Guatemala (Elías‐Gutiérrez, Kotov, & Garfias‐Espejo, 2006), and in other parts of North America (Penton et al., 2004). DNA barcoding in the present study also revealed that *Daphnia* species diversity on the Tibetan Plateau is much higher than previously thought (Chiang, 1963; Chiang & Chen, 1974; Chiang & Du, 1979; Chiang et al., 1983; Shen & Sung, 1964), doubling the described and provisional species number from 5 (*D. magna*,* D. tibetana*,* D. pulex*,* D. similoides*, and *D. dentifera*) to 10. Recently, an updated checklist of Chinese Cladocera was released based on literature analysis and our molecular data (Xiang et al., 2015). Approximately 19 species of *Daphnia* are now found in China. At least 10 of these species occur on the Tibetan Plateau, with some species such as *D*. cf. *himalaya*,* D*. cf. *tenebrosa*,* D. longispina,* and *D. pulicaria* being the first records identified by molecular data. Morphological similarity in some clades was the cause for hidden species diversity. Alternatively, species in the *D. longispina* clade show strong morphological plasticity (Laforsch & Tollrian, 2004; Petrusek, Tollrian, Schwenk, Haas, & Laforsch, 2009) compounded by the possibility of hybridization and introgression (Ishida et al., 2011; Keller, Wolinska, Tellenbach, & Spaak, 2007; Schwenk & Spaak, 1995). Morphology‐based taxonomy is insufficient for distinguishing the underlying genetic units. A lack of investigation has long delayed an appreciation of the diversity of *Daphnia* in Tibet. Only recently have studies begun to reveal the region's hidden species (Ma et al., 2015) and the impact of environmental change on cladoceran species richness and composition (Lin et al., 2017).

The genomic region of the COI gene sequence is used not only in DNA barcoding (Costa et al., 2007; Hebert, Cywinska, et al., 2003), but also in detecting speciation. The level of sequence divergence between congeneric species of crustaceans averaged 17.16%, the highest value so far in animals. As a comparison, congeneric species of lepidopterans show just 6.1% variation (Hebert, Cywinska, et al., 2003), birds 7.93% (Hebert et al., 2004), and fishes 9.93% (Ward et al., 2005). Congeneric divergences in *Daphnia* are reported by Costa et al. (2007) to be extremely high at 13.18%–30.65%, which is supported by our data (9.25%–30.71%; the highest divergence being between *D*. cf. *tenebrosa* and *D. magna*). The average interspecific divergence between *Daphnia* species on the Tibetan Plateau of 25.23% is similar to that reported in Argentina (25.28%, Adamowicz, Hebert, & Marinone, 2004), but significantly higher than values reported from Churchill, Canada (14.1%, Jeffery et al., 2011). Difference in average interspecific divergence may be related to species richness: 10 congeneric species occurred in Tibet and 11 South American endemics in Argentina, against only five species in the Churchill region.

The level of intraspecific variation in crustaceans averaged 0.69%, a value that is slightly higher than those reported in other groups (most range from 0.25% to 0.30%). High intraspecific variations in our study were found in *D. tibetana* (1.60%) and *D. pulicaria* (1.72%). We collected one population of *D. tibetana* and *D. pulicaria* from Zhaling Lake, which is more than 1,600 km away from the other investigated lakes, indicating the elevated divergence values came from Zhaling Lake samples. Possibly the high values reflect limited gene flow between the two species caused by physical barriers such as mountains that separate the Tibetan lakes from Zhaling Lake, followed by adaptation to local environmental pressure in the lake.

### 4.2 Biogeographic patterns of *Daphnia* on the Tibetan Plateau

Six of the 10 taxonomic units from DNA taxonomy in our study matched those determined by morphological taxonomy. However, our analysis distinguished three clades each for the *D. longispina* (*D. galeata*,* D. dentifera*, and *D. longispina*) and *D. pulex* (*D. pulex*,* D*. cf. *tenebrosa,* and *D. pulicaria*) complexes. Distributions of *Daphnia* species on the Tibetan Plateau were mostly nonoverlapping, with the exception of *D*. cf. *tenebrosa*,* D. pulex*, and *D. similoides* (Figure 1). *Daphnia tibetana* was the most common species in our investigation, being present in 12 of 28 water bodies without fish predators. *Daphnia tibetana* is endemic to the Tibetan Plateau, previously recorded as *Daphniopsis tibetana* Sars, 1903 (Chiang & Du, 1979). Glagolev (1983) and Benzie (2005) regard *Daphniopsis* as a junior synonym of *Daphnia*. There has long been confusion about the status of *D. tibetana* and *D. fusca,* but *D. fusca* was absent from our samples. *Daphnia tibetana* is distinguished from *D. fusca* by having rounded rather than angled fornices, combs on the postabdominal claws that are not strongly differentiated, fewer anal spines, no spines on the carapace margins, a sinuate anterior margin to the head in some specimens, no dorsal ridge and a short rostrum, and two well developed postabdominal processes (Benzie, 2005). Previous investigations showed that *D. tibetana* is a halobiont, living at more than 4,000 m in hyposaline to mesosaline lakes in Tibet, Mongolia, and India. Our sample area covered a large geographical range (>2,200,000 km^2^) containing different habitats located at latitudes ranging from 2,700 m to about 5,000 m asl. Water temperatures at which the samples were collected ranged from 2°C to 20°C, salinity varied from 9 to 35 g/L, and pH ranged from 9.0 to 10.4 (Zhao, Wang, Zheng, Zhao, & Wang, 2002). However, a recent study (Lin et al., 2017) reported an even wider salinity range (6.4–46.2 g/L) for *D. tibetana*. The northernmost population in our investigation was found in Sugan Lake, a closed inland ecosystem located to the north of Qaidam Basin at an altitude of 2,796 m, the lowest known altitude where *D. tibetana* occurs. Sugan Lake is situated more than 1,200 km from where other *D. tibetana* are found*,* and is an important habitat for migratory birds. Thirty‐eight bird species have been observed in the wetlands around this lake (Bao, Zhang, Liu, Song, & Zhao, 2007). Bird migration is the most likely explanation for *D. tibetana* presence in the lake, although research is required to identify which birds are the vectors involved in long‐distance dispersal of the cladoceran.

The *Daphnia longispina* complex was found in eight water bodies from the westernmost Ngari Prefecture to Lhasa River. The lineage of *D. longispina* was found in Bangong co and Lang co, Ngari area, western Tibet, near to the recently confirmed easternmost locality of Pamir Mountains (Möst et al., 2013). Its distribution extends to western China. In contrast to previous taxonomic studies based solely on morphology (Chiang & Du, 1979), our finding suggests widespread presence of the *D. longispina* complex across the whole of China. *Daphnia galeata* was reported from northern and southwestern China and from the Yangtze Basin (Xu, 2013), and presumably coexists with *D. longispina* in east China. But because *D. galeata*,* D. dentifera* and *D. longispina* have similar morphologies, classification errors are likely. Thus, *D. longispina* phenotypes reported from the 1970s are suspect and may have been incorrectly identified (Wei et al., 2015). Moreover, *D. longispina* has been documented to typically occur in alpine oligotrophic lakes (Hamrová, Krajicek, Karanovic, Černý, & Petrusek, 2012; Ventura et al., 2014). The absence of *D. longispina* phenotypes in lowland China may be related to the rareness of oligotrophic lakes in eastern China caused by eutrophication. Our molecular results also confirm recent reports on the *D. longispina* complex across China, in which *D. galeata* was the only lineage found in the eastern low‐altitude plain, whereas *D. dentifera* dominated in lakes of the Tibetan Plateau and *D. longispina* was absent from east China (Ma et al., 2015). *Daphnia* cf. *himalaya* is especially intriguing, as it was found in two permanent lakes and one temporary pond along the Nyenchenthanglha Mountain in the center of the Tibetan Plateau. The morphology of *D*. cf. *himalaya* in our collection is similar to the dark‐pigmented *Daphnia*‐like species described by Manca, Martin, Peñalva‐Arana, and Benzie (2006) and named *Daphnia himalaya* from the Khumbu Region in Nepal. However, the absence of males in our samples suggests further investigation is needed.

## 5 CONCLUSION

Our study is the first to use DNA barcoding as a tool to delineate species and their distribution pattern in Tibet. The technique revealed 10 described and provisional species of *Daphnia* on the Tibetan Plateau. This diversity is double of that shown by previous checklists. The sequence divergence among *Daphnia* was high and varied between 9.25% and 30.71%. Two species, *D. tibetana* and *D*. cf. *himalaya*, are endemic to the plateau and the Himalayas. The hygrophile *D. tibetana,* presumed to be the result of local speciation, was the most common species that was found in 12 hyposaline to mesosaline lakes. Our study is the first time to confirm the presence of the *D. longispina* lineage in western Tibet.

## CONFLICT OF INTEREST

None declared.

## AUTHOR CONTRIBUTIONS

L. Xu sampled the zooplankton samples, analyzed the data, and wrote the manuscript. Q. Lin performed the research, sampled the zooplankton samples, and wrote the manuscript. S. Xu, Y. Gu, J. Hou, and Y. Liu sampled the zooplankton samples. H. J. Dumont wrote the manuscript. B.‐P. Han designed the research, supported the fieldwork, and wrote the manuscript.

## DATA ACCESSIBILITY

DNA sequences: GenBank accessions: MG544001–MG544093.
