# Supplementary material for: Daphnia diversity on the Tibetan Plateau measured by DNA taxonomy
Source: Ecol Evol. 2018 Apr 24;8(10):5069–78. doi: 10.1002/ece3.4071 (PMC5980554; doi:10.1002/ece3.4071)
Supplement: Supplementary file 1 [file ECE3-8-5069-s001.docx]

| Morphological type | Accession numbers | Collection location | References or collector | Haplotype code |
| --- | --- | --- | --- | --- |
| *Daphnia longispina* | EF37586 | Switzerland: Great St. Bernard pass | Petrusek et al. (2008) | D. longispina1 |
| *Daphnia longispina* | EF375860 | Germany: Lake Constance | Petrusek et al. (2008) | D. longispina2 |
| *Daphnia dentifera* | KM555366 | China: Tibet | Ma et al. (2014) | D. dentifera1 |
| *Daphnia dentifera* | KM555369 | China: Tibet | Ma et al. (2014) | D. dentifera2 |
| *Daphnia galeata* | KY700828 | Australia: Loveday Bay, L. Alexandrina | Karabanov et al. (2017) | D. galeata1 |
| *Daphnia galeata* | EF375867 | Netherlands: Lake Tjeukemeer | Petrusek et al. (2008) | D. galeata2 |
| *Daphnia magna* | GU680597 | Canada: Manitoba | Carr et al. (2010) | D. magna1 |
| *Daphnia magna* | KJ461675 | Anhui, China | Geng et al. (2014) | D. magna2 |
| *Daphnia pulex* | KJ461674 | Anhui, China | Geng et al. (2014) | D. pulex1 |
| *Daphnia pulex* | GU595192 | Japan: pond near Hibara Park | Kotov and Taylor (2010) | D. pulex2 |
| *Daphnia pulicaria* | JN233925.1 | Canada: Manitoba, Churchill | Jeffery et al. (2011) | D. pulicaria |
| *Daphnia similoides* | KF960111 | China: Heilongjiang Province, Longhe Farm | Huang et al. (2014) | D. similoides |
| *Daphnia tenebrosa* | HM400735 | Canada: Manitoba | Lim (2010) | D. tenebrosa1 |
| *Daphnia tenebrosa* | JN233926 | Canada: Manitoba, Churchill | Jeffery et al (2011) | D. tenebrosa2 |
| *Daphnia tibetana* | KX890200 | China: Tibet | Zhao et al. (2016) | D. tibetana1 |
| *Daphnia tibetana* | KX890200 | China: Tibet | Zhao et al. (2016) | D. tibetana2 |

Appendix table

The information of *Daphnia* COI sequences which deposited in Genbank
